# Associations of Three Well-Characterized Polymorphisms in the *IL-6* and *IL-10* Genes with Pneumonia: A Meta-Analysis

**DOI:** 10.1038/srep08559

**Published:** 2015-02-24

**Authors:** Hong Chen, Ning Li, Huanying Wan, Qijian Cheng, Guochao Shi, Yun Feng

**Affiliations:** 1 Department of Respiration, Ruijin Hospital, School of Medicine, Shanghai Jiao Tong University, Shanghai, China; 2 Department of Respiration, Ruijin North Hospital, School of Medicine, Shanghai Jiaotong University, Shanghai, China

## Abstract

Published data on the associations between three well-characterized polymorphisms in the interleukin 6 and 10 (*IL-6* and *IL-10*) genes and the risk of pneumonia are inconclusive. A meta-analysis was performed to derive a more precise estimate. The electronic databases MEDLINE (Ovid) and PubMed were searched from the earliest possible year to May 2014. A total of 9 articles met the criteria, and these included 3460 patients with pneumonia and 3037 controls. The data were analyzed with RevMan software, and risk estimates are expressed as odds ratios (ORs) and 95% confidence intervals (95% CIs). Analyses of the full data set failed to identify any significant association of pneumonia risk with the *IL-6* gene -174C allele (OR = 1.00; 95% CI: 0.98–1.03), the *IL-10* gene -592C allele (OR = 1.20; 95% CI: 0.95–1.52), or the *IL-10* gene -1082A allele (OR = 1.21; 95% CI: 0.99–1.49). In a subgroup analysis by pneumonia type, ethnicity, sample size and quality score, no significantly increased risk of pneumonia was found for individuals carrying the *IL-6* gene -174C allele. There was a low probability of publication bias, as reflected by the fail-safe number. This meta-analysis suggests that there is no significantly increased risk of pneumonia associated with previously reported *IL-6* and *IL-10* polymorphisms.

Pneumonia is a major cause of morbidity and mortality worldwide^1^. The strength of the immune response in humans is associated with the occurrence and severity of this disease. Cytokines released by inflammatory cells are important for the host immune response. Major pro-inflammatory cytokines include tumor necrosis factor-α (TNF-α) and interleukin 6 (IL-6). Interleukin 10 (IL-10) is considered to be the most important anti-inflammatory cytokine. Interleukin genes may play a key role in the pathogenesis of pneumonia^2^. The relationship between pneumonia and polymorphisms of interleukin genes has been studied extensively.

The importance of IL-6 in many physiological and pathological processes, particularly in the inflammatory response, has been reported^3^. In patients with unilateral pneumonia, Dehoux and colleagues found that the IL-6 level in bronchoalveolar lavage fluid obtained from the infected lung was significantly higher than that in the uninfected side or in the plasma. Waage et al. showed that elevated plasma levels of IL-6 are associated with high mortality^4^. In addition, several studies have reported increased IL-10 levels in the blood of patients with severe sepsis or septic shock^5^.

Several polymorphisms in the promoter regions of *IL-6* and *IL-10*, such as *IL-6* -174G/C (rs1800795), *IL-6* -572G/C (rs1800796), *IL-10* -592C/A (rs1800872), and *IL-10* -1082G/A (rs1800896), have been identified. Previous studies have reported associations between *IL-6* and *IL-10* polymorphisms and the risk of pneumonia^6^,^7^,^8^,^9^,^10^,^11^,^12^,^13^,^14^. Although exhaustive association studies have been undertaken to address this issue, no definitive conclusion has yet been reached, and the results have been irreproducible. To generate more information, we carried out a meta-analysis of all of the available case-control studies to investigate the association of genetic polymorphisms of *IL-6* and *IL-10* with the risk of pneumonia. The selection of polymorphisms under investigation was straightforward if three or more unduplicated studies were available for a certain polymorphism of *IL-6* and *IL-10* genes.

## Methods

### Ethics

The study protocol was approved by the Coordinating Ethics Committee of Ruijin Hospital, and the study methods were carried out in accordance with the approved guidelines.

### Search strategy for the identification of studies

We searched PubMed and MEDLINE (Ovid) for articles published before May 2014. The subject terms included either interleukin-6 (or *IL-6*) or interleukin-10 (or *IL-10*) and pneumonia. The search results were expressed using Boolean operators: ((interleukin-6) OR *IL-6* OR (interleukin-10) OR *IL-10*) AND (pneumonia) AND (gene OR polymorphism OR alleles OR variants)) AND English [Language].

### Inclusion/exclusion criteria

Our analyses were restricted to articles that fulfilled the following inclusion criteria (with all having to be satisfied): 1) investigation of the association between genetic polymorphisms of the *IL-6* and *IL-10* genes and pneumonia among unrelated subjects; 2) genotypes of the examined polymorphisms were tested in a validated sample size; 3) a case-control study design; and 4) sufficient information on the genotypes or alleles of the examined polymorphisms to allow estimation of the odds ratio (OR) and its corresponding 95% confidence interval (95% CI). Articles were excluded (with one condition being sufficient to do so) if they investigated the progression or severity of pneumonia, phenotype modification, or response to treatment or survival, as well as if they were conference abstracts, case reports/series, editorials, review articles, or non-English articles. If there were multiple publications from the same study group, the most complete and recent results were used. The search results were limited to articles published in English and studies performed in humans.

### Data extraction

Two reviewers (C.H. and L.N.) independently assessed all potentially relevant studies and reached a consensus on all items. In cases of disagreement, a third author provided an assessment. The following data were collected from each study: first author, year of publication, ethnicity, study design, diagnostic criteria, baseline characteristics of the study population, total number of cases and controls, and genotype distributions in cases and controls. After data extraction, discrepancies were adjudicated by discussion until a consensus was reached.

### Quality score assessment

The study quality was evaluated using a quality assessment score developed for genetic association studies by Thakkinstian and colleagues^15^. Total scores ranged from 0 (the worst) to 12 (the best). The criteria for the quality assessment of genetic associations between the *IL-6* gene C-174G polymorphism and pneumonia are described in Table S1.

### Statistical methods

The meta-analysis was calculated using Review Manager version 5.0.19 software, available at http://ims.cochrane.org/revman/download. The Hardy-Weinberg equilibrium was assessed using Pearson's χ^2^ test or Fisher’s exact test (SAS version 9.1.3, Institute Inc., Cary, NC, USA). The inconsistency index (I^2^) was used to quantify the presence of between-study heterogeneity, with statistical significance set at 0.1^16^. When the P value was >0.10, the pooled OR was calculated using the fixed-effects model; otherwise, a random-effects model was used. Sensitivity analyses were performed to look at more narrowly drawn subsets of the studies by removing an individual study or by removing studies with similar feature to assess their influence separately. Predefined subgroup analyses were performed a priori according to ethnicity (Caucasian or mixed), age (adult or pediatric), the pneumonia type (CAP or HAP), total sample size (<500 subjects or ≥500 subjects), or the quality score (score <11 or score ≥11).

Publication bias was assessed by the fail-safe number (N_fs_), with the significance set at 0.05 for each meta-comparison. Specifically, if the calculated N_fs_ value was smaller than the number of studies observed, the meta-analysis results might have publication bias. We calculated the N_fs_0.05 according to the formula N_fs_0.05 = (ΣZ/1.64) 2 − k, where k is the number of articles included.

## Results

### Study characteristics

**Figure 1:**
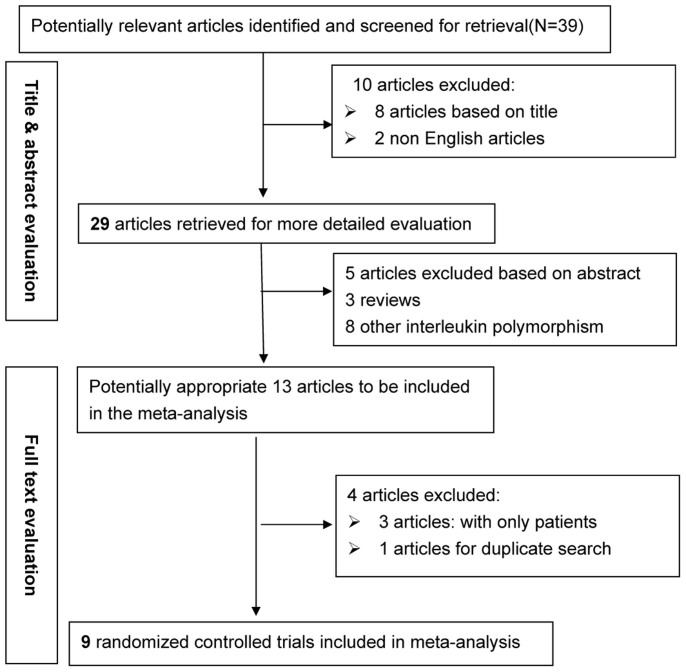
Flow diagram of the search strategy and study selection.

Based on the search strategy, our primary search produced 39 potentially relevant articles, of which 9 articles met the inclusion criteria^6^,^7^,^8^,^9^,^10^,^11^,^12^,^13^,^14^. In total, 3460 patients with pneumonia and 3037 controls were examined. The detailed selection process is presented in Figure 1. The details of each excluded study have been uploaded as a supplementary information file (Table S2). The baseline characteristics of the included studies are presented in Table 1.

Of these studies, 7 articles examined the association of the *IL-6* -174G/C polymorphism with pneumonia^6^,^7^,^8^,^9^,^10^,^11^,^12^. Because Salnikova LE et al. had published two articles on the same study group, we used the more recent result^10^,^17^. Three articles focused on the *IL-10* gene -592C/A polymorphism^8^,^11^,^13^, and 3 articles focused on the *IL-10* gene -1082G/A polymorphism^8^,^11^,^14^.

### Overall analyses

**Figure 2:**
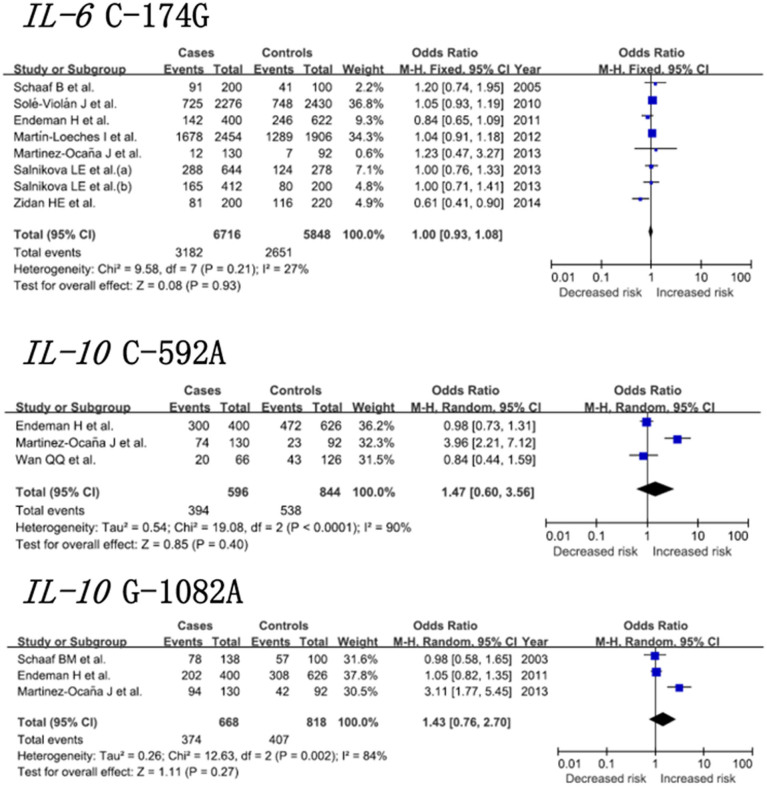
Pooled risk estimates of pneumonia for the *IL-6* gene C-174G, *IL-10* gene C-592A and *IL-10* gene G-1082A polymorphisms under the allelic model.

Figure 2 depicts the pooled risk estimates of developing pneumonia for the mutant alleles of the three *IL-6* and *IL-10* gene polymorphisms. Under a random-effects model, the analyses of the full data set failed to reveal any significant association of the *IL-6* -174C allele (OR = 1.00; 95% CI: 0.93–1.08), the *IL-10* -592C allele (OR = 1.20; 95% CI: 0.95–1.52), or the *IL-10* -1082A allele (OR = 1.43; 95% CI: 0.76–2.70) with risk of pneumonia. Sensitivity analyses were performed by excluding studies with controls not in HWE. The results show that the associations between the *IL-10* gene -592C and *IL-10* gene -1082A polymorphisms and pneumonia risk were not significantly altered.

### Subgroup analyses

In view of the number of included articles, subgroup analyses were undertaken only for the *IL-6* gene -174 C/G polymorphism, with regard to age, pneumonia type, ethnicity, sample size and quality score (table 2). The subgroup analysis stratified by age showed that no associations existed in adults (OR = 1.02, 95% CI: 0.95–1.11, p = 0.56). In the subgroup analysis of the type of pneumonia, no significantly increased risk of pneumonia was found for CAP (OR = 1.00, 95% CI: 0.93–1.08, p = 0.93). The subgroup analysis stratified by ethnicity showed that no association existed in Caucasians (OR = 1.02, 95% CI: 0.95–1.11, p = 0.56). With regard to sample size, no significance was reached in large studies (the total sample size ≥500 participants) or in small studies (the total sample size <500 participants). With regard to quality score, there were no significant findings observed under any of the four genetic models in low-quality studies (quality score <11) or in high-quality studies (quality score ≥11).

### Publication bias

**Figure 3:**
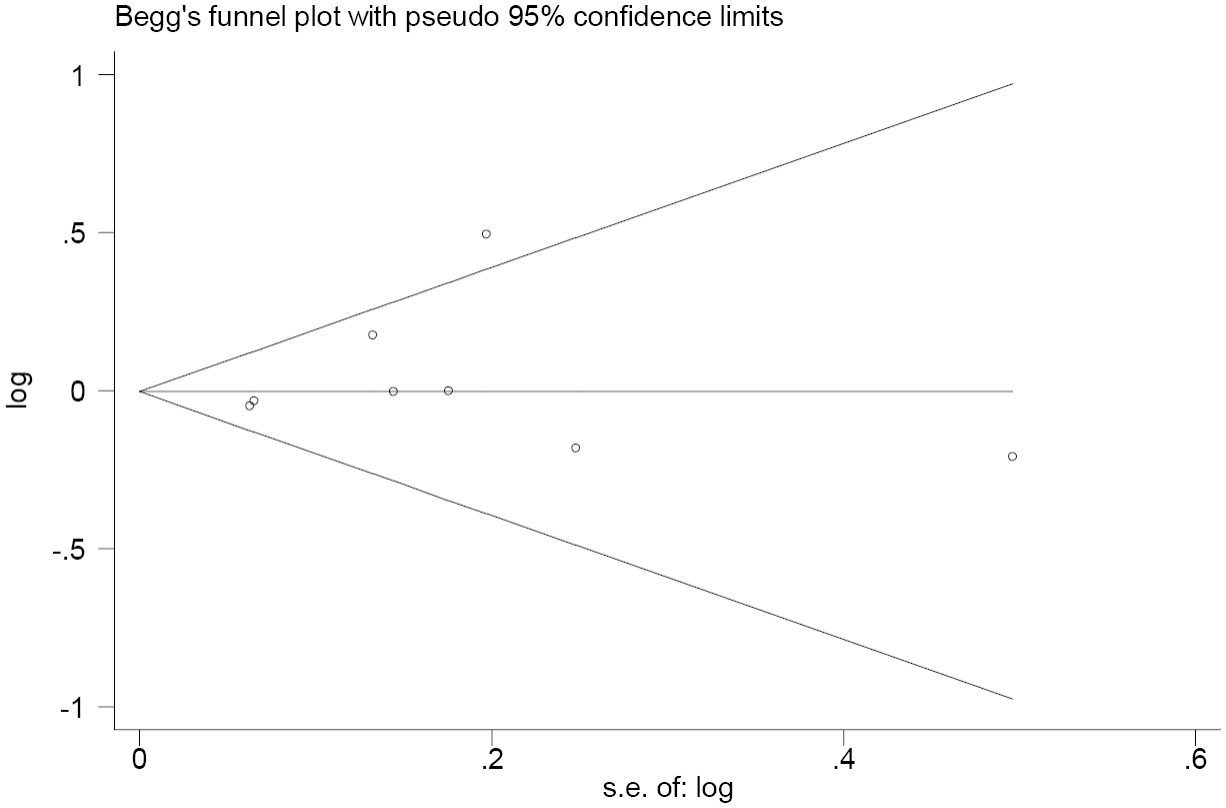
Begg’s funnel plot of the Egger test for publication bias of the *IL-6* gene -174 C/G polymorphism and pneumonia risk analysis. The horizontal line in the funnel plot indicates the fixed-effects summary estimates, and the sloping lines indicate the expected 95% CI for a given standard error. The sizes of the circles in each plot are positively proportional to the sample sizes of each study.

The N_fs_ values were calculated to assess the potential existence of publication bias. At a significance level of 0.05, the N_fs_0.05 values were consistently greater than the number of studies included in this meta-analysis for all polymorphisms under investigation. In the analysis of the *IL-6* gene -174 C/G polymorphism and pneumonia risk, the resultant symmetrical funnel shape was consistent with the absence of publication bias in the funnel plot for contrasts of C versus G (P-Egger test = 0.475) (Figure 3).

## Discussion and conclusions

In this study, we sought to investigate the association of *IL-6* and *IL-10* genetic polymorphisms with pneumonia risk by conducting a meta-analysis of studies reported in English journals, and we included 9 articles covering 6497 subjects. This meta-analysis demonstrated an absence of association between the *IL-6* gene C-174G, *IL-10* gene C-592A and *IL-10* gene G-1082A polymorphisms and pneumonia risk. Moreover, a subgroup analysis indicated no significantly increased risk of CAP among adults. To the authors’ knowledge, this is the first meta-analysis investigating the association of the *IL-6* gene C-174G, *IL-10* gene C-592A and *IL-10* gene G-1082A genetic polymorphisms with pneumonia risk.

Results from our meta-analysis show a lack of association between *IL-6* and *IL-10* gene polymorphisms and pneumonia risk. Although many studies have reported that the allele *IL-*6-174C is associated with increased IL-6 secretion^18^,^19^, our study did not find such an association. Some studies have shown that *IL-10*-1082 G is associated with increased secretion of IL-10 in chronic hepatitis B virus infection^20^ and clinical malaria^21^, although no significantly increased risk of pneumonia was found. There are two potential reasons for the results. First, because of the complex nature of pneumonia, it is unlikely that a single nucleotide polymorphism in a single gene would be associated with an increased risk of pneumonia or mortality, without a contribution from other polymorphic susceptibility genes. Second, other factors, such as age, pathogenic organism, medical treatment, and nutrient status, can also influence the development or the prognosis of pneumonia. Three studies have reported on the association of *IL-6* -174 GG genotype with systemic inflammatory response syndrome (SIRS) and mortality from pneumonia^7^,^9^,^22^. However, the studies used different standards to extract data, so a meta-analysis could not be conducted. This issue needs to be further studied. Paats et al. found that IL-6 and IL-10 play important roles in CAP. They showed that the level of IL-6 was significantly increased in the bronchoalveolar lavage fluid of CAP patients compared with healthy individuals and that serum levels of IL-6 and IL-10 were significantly higher in patients with severe CAP than in those with non-severe CAP or healthy individuals^23^. Kwan J and colleagues found that *IL-6* is independently associated with stroke-associated infection and may be a key biomarker^24^.

We also carried out subgroup analyses by age, pneumonia type, ethnicity, sample size and quality score. For ethnicity, our results showed no significant increase in risk of pneumonia among Caucasians. Subgroup analyses also did not detect a significant association between *IL-6* -174 and pneumonia risk in adults with CAP. We also found that no association existed between *IL-6* -174 and pneumonia for any sample size or quality score.

This study has several limitations. First, only published studies in English were included; it is possible that some relevant published or unpublished studies with null results were missed, which might have biased the results. Second, owing to the relatively small number of eligible studies, we were unable to perform further subgroup analyses, such as those by ethnicity or gender, because of limited data. Third, because the data extracted from the primary publications were insufficient, we could not assess the effects of the *IL-6* -572G/C, 1753C/G, 2954G/C, *IL-10* -819 C/T and interleukin-1 receptor antagonist intron 2^25^ polymorphisms on pneumonia risk. Fourth, the statistical heterogeneities of the effects of *IL-10* C-592A and *IL-10* G-1082A were significant in our meta-analyses, likely because only three studies of these polymorphisms were included in the meta-analysis and these studies were conducted in different countries and had different sample sizes. Finally, the lack of original data in the eligible studies limited the evaluation of the effects of gene-gene interactions in pneumonia. Therefore, the jury remains out before the eventual truth prevails. We minimized the likelihood of bias by creating a detailed protocol before initiating our study, performing a meticulous search for publications, and using explicit methods for publication selection, data extraction, and analysis.

In conclusion, our results suggest that *IL-6* and *IL-10* gene polymorphisms are not associated with the risk of pneumonia. Future studies with large sample sizes and more ethnic groups are needed to confirm our findings. Moreover, other interleukin polymorphisms and gene-gene interactions should also be considered in future studies.

## Author Contributions

Conception and design of the experiments: Y.F. and G.C.S. Execution of the experiments: H.C. and N.L. Analysis of the data: Y.F., H.Y.W. and Q.J.C. Contribution of reagents/materials/analytical tools: Y.F. and G.C.S. Composition of the manuscript: Y.F. and Q.J.C.

## Supplementary Material

Supplementary Information Table S1,S2

## Figures and Tables

**Table 1:** The baseline characteristics of all study populations in the meta-analysis

Study	Year	Ethnicity	Pneumonia	Age	Quality score	Sample size		Allele distributions				
			type	group		controls	cases	controls		cases		Characteristics
**IL-6 -174 C/G**								**C**	**G**	**C**	**G**	
Schaaf B et al.	2005	German	CAP	adult	9	50	100	41	59	91	109	The controls were sex- and age-matched healthy volunteers.
							^a^P_HWE_	0.813				
Solé-Violán J et al.	2010	Spanish	CAP	adult	11	1215	1138	748	1682	725	1551	Cases (age: 49.04 ± 17.40, 43.1% women)
							P_HWE_	0.289				
Endeman H et al.	2011	Dutch	CAP	adult	8	311	200	246	376	142	258	The controls were sex- and age-matched healthy volunteers.
							P_HWE_	0.878				
Martín-Loeches I et al.	2012	Spanish	CAP	adult	10	953	1227	1289	617	1678	776	Controls (age: 43.95 ± 16.3,41.16% males); Cases (age: 59.9 ± 17.3, 34.6% women)
							P_HWE_	0.752				
Salnikova LE et al.(a)	2013	Russian	CAP	adult	11	139	322	124	154	288	356	Controls (130 males and 11 females; age range: 18–52 years, mean age: 29 years); Cases (307 males and 27 females; age range: 18–55 years, mean age: 27 years)
							P_HWE_	0.052				
Salnikova LE et al.(b)	2013	Russian	HAP	adult	11	100	206	80	120	165	247	Controls (83 males and 22 females; age range: 19–93 years, mean age: 41 years); Cases (176 males and 40 females; age range: 18–82 years, mean age: 43 years).
							P_HWE_	0.096				
Martinez-Ocaña J et al.	2013	Mexican	CAP	adult	7	46	65	7	85	12	118	Controls (age: 35.7 ± 11.8, 51% males); Cases (age: 35.3 ± 19.1, 49% males). Cases are influenza A(H1N1)pdm09-infected patients
							P_HWE_	0.576				
Zidan HE et al.	2014	Egyptian	CAP	pediatric	11	110	100	116	104	81	119	Cases (52 males and 48 females; age range: 60 days–13 years, mean age: 2.1years).
							P_HWE_	0.323				
**IL-10 -592 C/A**								**C**	**A**	**C**	**A**	
Endeman H et al.	2011	Dutch	CAP	adult		313	200	472	154	300	100	The controls were sex- and age-matched healthy volunteers.
							P_HWE_	0.132				
Wan QQ et al.	2013	Chinese	CAP	adult		63	33	43	83	20	46	Cases (age: 39.3 ± 10.2, 23 males and 10 females).
							P_HWE_	0.710				
Martinez-Ocaña J et al.	2013	Mexican	CAP	adult		46	65	23	69	69	61	Controls (age: 35.7 ± 11.8, 51% males); Cases (age: 35.3 ± 19.1, 49% males). Cases are influenza A(H1N1)pdm09-infected patients
							P_HWE_	0.024				
**IL-10 -1082 C/A**								**G**	**A**	**G**	**A**	
Schaaf BM et al.	2003	German	CAP	adult		50	69	43	57	60	78	The controls were sex- and age-matched healthy volunteers.
							P_HWE_	0.301				
Endeman H et al.	2011	Dutch	CAP	adult		313	200	318	308	198	202	The controls were sex- and age-matched healthy volunteers.
							P_HWE_	0.125				
Martinez-Ocaña J et al.	2013	Mexican	CAP	adult		46	65	50	42	36	94	Controls (age: 35.7 ± 11.8, 51% males); Cases (age: 35.3 ± 19.1, 49% males). Cases are influenza A(H1N1)pdm09-infected patients
							P_HWE_	0.006				

**Table 2:** Summary of various comparative results

Genetic model		Overall or subgroup	Study number(n)	Participants (n)	OR (95% CI)	Z	P	I^2^ (%)	P_het_
**IL-6 -174 C/G**									
C *vs* G		All	8	12,564	1.00 (0.93, 1.08)	0.08	0.93	27	0.21
	Age	All excluding pediatric	7	12,144	1.02 (0.95, 1.11)	0.59	0.56	0	0.81
	Pneumonia type	All excluding HAP	7	11,952	1.00 (0.93, 1.08)	0.08	0.93	37	0.14
	Ethnicity	Caucasians	7	12,144	1.02 (0.95, 1.11)	0.59	0.56	0	0.81
	Quality score	≥11	4	6660	1.00 (0.90, 1.11)	0.00	1.0	56	0.08
		<11	4	5904	1.01 (0.90, 1.12)	0.12	0.91	0	0.44
	Sample size	≥500	3	10088	1.02 (0.94, 1.11)	0.46	0.64	16	0.30
		<500	5	2476	0.93 (0.79, 1.11)	0.77	0.44	38	0.17
CC *vs* GG		All	8	3547	1.00 (0.84, 1.18)	0.02	0.98	40	0.12
	Age	All excluding pediatric	7	3452	1.05 (0.88, 1.24)	0.54	0.59	0	0.73
	Pneumonia type	All excluding HAP	7	3378	0.98 (0.83, 1.17)	0.21	0.84	47	0.10
	Ethnicity	Caucasians	7	3452	1.05 (0.88, 1.24)	0.54	0.59	0	0.73
	Quality score	≥11	4	1821	1.01 (0.80, 1.21)	0.08	0.93	62	0.05
		<11	4	1686	0.98 (0.77, 1.26)	0.13	0.90	2	0.36
	Sample size	≥500	3	2886	1.01 (0.84, 1.22)	0.13	0.89	0	0.41
		<500	5	661	0.94 (0.65, 1.37)	0.32	0.75	63	0.04
CG *vs* GG		All	8	4733	0.95 (0.84, 1.07)	0.90	0.37	33	0.16
	Age	All excluding pediatric	7	4564	0.96 (0.85, 1.09)	0.65	0.52	33	0.18
	Pneumonia type	All excluding HAP	7	4481	0.97 (0.86, 1.11)	0.40	0.69	7	0.38
	Ethnicity	Caucasians	7	4564	0.96 (0.85, 1.09)	0.65	0.52	33	0.18
	Quality score	≥11	4	2901	0.94 (0.81, 1.09)	0.82	0.41	68	0.03
		<11	4	1832	0.96 (0.77, 1.19)	0.39	0.69	0	0.77
	Sample size	≥500	3	3709	1.02 (0.89, 1.17)	0.23	0.82	0	0.50
		<500	5	1024	0.71 (0.54, 0.94)	2.42	0.02	0	0.41
CC + CG *vs* GG		All	8	6282	0.96 (0.86, 1.08)	0.60	0.55	26	0.22
	Age	All excluding pediatric	7	6072	0.99 (0.88, 1.11)	0.23	0.82	0	<0.43
	Pneumonia type	All excluding HAP	7	5976	0.98 (0.87, 1.11)	0.28	0.78	23	0.25
	Ethnicity	Caucasians	7	6072	0.99 (0.88, 1.11)	0.23	0.82	0	0.43
	Quality score	≥11	4	3330	0.96 (0.84, 1.11)	0.50	0.62	61	0.05
		<11	4	2952	0.97 (0.79, 1.19)	0.33	0.74	0	0.60
	Sample size	≥500	3	5044	1.02 (0.90, 1.16)	0.29	0.77	0	0.37
		<500	5	938	0.77 (0.77, 1.01)	1.91	0.06	4	0.39
CC *vs* CG + GG		All	8	6282	1.05 (0.93, 1.19)	0.79	0.43	47	0.08
	Age	All excluding pediatric	7	6072	1.08 (0.95, 1.23)	1.37	0.22	8	0.37
	Pneumonia type	All excluding HAP	7	5976	1.03 (0.90, 1.17)	0.44	0.66	41	0.13
	Ethnicity	Caucasians	7	6072	1.08 (0.95, 1.23)	1.23	0.22	8	0.37
	Quality score	≥11	4	3330	1.08 (0.88, 1.34)	0.75	0.45	69	0.02
		<11	4	2952	1.03 (0.88, 1.21)	0.42	0.67	0	0.49
	Sample size	≥500	3	5044	1.03 (0.90, 1.18)	0.45	0.65	0	0.56
		<500	5	1238	1.17 (0.85, 1.62)	0.96	0.34	69	0.02
**IL-10 -592 C/A**									
C *vs* A		All	3	1440	1.20 (0.95, 1.52)	0.85	0.40	90	0.0001
		All in HWE	2	1218	0.95 (0.73, 1.24)	0.35	0.72	0	0.67
CC *vs* AA		All	3	441	1.38 (0.79, 2.42)	1.14	0.25	84	0.002
		All in HWE	2	370	0.59 (0.29, 1.17)	0.51	0.13	0	0.76
CA *vs* AA		All	3	396	0.74 (0.46, 1.20)	1.21	0.23	0	0.45
		All in HWE	2	311	0.73 (0.41, 1.31)	1.06	0.29	37	0.21
CC + CA *vs* AA		All	3	720	1.02 (0.65, 1.60)	0.10	0.92	60	0.08
		All in HWE	2	609	0.72 (0.41, 1.27)	1.13	0.26	0	0.38
CC *vs* CA + AA		All	3	720	1.38 (1.00, 1.91)	0.62	0.54	78	0.01
		All in HWE	2	609	1.05 (0.74, 1.49)	0.27	0.79	13	0.28
**IL-10 -1082 G/A**									
A *vs* G		All	3	1486	1.43 (0.72, 2.70)	1.11	0.27	84	0.002
		All in HWE	2	1264	1.04 (0.83, 1.30)	0.33	0.74	0	0.81
AA *vs* GG		All	3	384	1.35 (0.89, 2.03)	1.42	0.16	77	0.01
		All in HWE	2	327	1.09 (0.70, 1.69)	0.39	0.70	0	0.87
AG *vs* GG		All	3	532	1.38 (1.00, 1.91)	1.07	0.28	0	0.51
		All in HWE	2	462	0.81 (0.54, 1.20)	1.06	0.29	25	0.25
AA + AG *vs* GG		All	3	743	0.97 (0.69, 1.37)	0.16	0.87	19	0.29
		All in HWE	2	632	0.89 (0.62, 1.29)	0.62	0.54	0	0.49
AA *vs* AG + GG		All	3	743	1.65 (1.19, 2.28)	1.29	0.20	87	0.0005
		All in HWE	2	632	1.21 (0.79, 1.83)	0.87	0.38	17	0.27

## Notes

This study was supported by the National Natural Science Foundation of China (81201837).
