# Supplementary material for: Associations of Three Well-Characterized Polymorphisms in the IL-6 and IL-10 Genes with Pneumonia: A Meta-Analysis
Source: Sci Rep. 2015 Feb 24;5:8559. doi: 10.1038/srep08559 (PMC4338428; doi:10.1038/srep08559)
Supplement: Supplementary Information — Table S1,S2 [file srep08559-s1.pdf]

Association of three Well-Characterized Polymorphisms in *IL -6* and *IL -10* Genes with Pneumonia: A Meta-Analysis

Hong Chen[1]*, Ning Li[1]*, Huanying Wan[1], Qijian Cheng[2], Guochao Shi[1], Yun Feng[1]
[1]Department of Respiration, Ruijin Hospital, School of Medicine, Shanghai Jiao Tong University, Shanghai, China
[2]Department of Respiration, Ruijin North Hospital, School of Medicine, Shanghai Jiaotong University, Shanghai, China
* These authors contributed equally to this work.
Correspondence and requests for materials should be addressed to G.C.S.(shiguochao@hotmail.com) or Y.F.(fy01057@163.com) or Q.J.C (chengqijian@yahoo.cn)

Supplementary Table S1. Criteria for quality assessment of genetic associations of the *IL-6* gene C-174G polymorphism with pneumonia risk

Criteria	Quality score
Representativeness of cases	
A. Consecutive/randomly selected from case population with clearly defined random frame	2
B. Consecutive/randomly selected from case population without clearly defined random frame or with extensive inclusion criteria	1
C. Method of selection not described	0
Representativeness of controls	
D. Controls were consecutive/randomly drawn from the same area (ward/community) as cases with the same criteria	2
E. Controls were consecutive/randomly drawn from a different area than cases	1
F. Not described	0
Ascertainment of pneumonia cases	
G. Clearly described objective criteria for diagnosis of pneumonia	1
H. Not described	0
Ascertainment of controls	
I. Clinical examinations were performed on controls to prove that controls did not have pneumonia	2
J. Article merely stated that controls were subjects who did not have pneumonia; no proof provided	1
K. Not described	0
Ascertainment of genotyping examination	
L. Genotyping done under "blind" conditions	1

M.	Unblended or not mentioned	0
Test for Hardy-Weinberg equilibrium		
N.	Hardy-Weinberg equilibrium in control group	2
O.	Hardy-Weinberg disequilibrium in control group	1
P.	Hardy-Weinberg equilibrium not checked	0
Association assessment		
Q.	Assessed association between genotypes and pneumonia with appropriate statistic and adjusting confounders	2
R.	Assessed association between genotypes and pneumonia with appropriate statistic without adjusting confounders	1
S.	Inappropriate statistic used	0

Supplementary Table S2. Excluded studies of IL-6, IL-10 gene polymorphisms and pneumonia risk.

exclusion criteria	Number	Titles of Excluded studies
based on title	8	Relationship between cytokine gene polymorphisms and risk of postoperative pneumonia with esophageal cancer.<br>Sex and inflammation in respiratory diseases: a clinical viewpoint.<br>Human parainfluenza virus serotypes differ in their kinetics of replication and cytokine secretion in human tracheobronchial airway epithelium.<br>Pulse methylprednisolone therapy in type 3 adenovirus pneumonia with hypercytokinemia.<br>Cytokine gene polymorphisms moderate illness severity in infants with respiratory syncytial virus infection.<br>A human origin type II strain of Toxoplasma gondii causing severe encephalitis in mice.<br>Pulse methylprednisolone therapy in type 3 adenovirus pneumonia with hypercytokinemia.<br>The NLRP3 inflammasome is differentially activated by pneumolysin variants and contributes to host defense in pneumococcal pneumonia.
non English articles	2	TNF-α, IL-10, and eNOS gene polymorphisms in patients with influenza A/H1N1 complicated by pneumonia.<br>Genetic markers of predisposition to infectious complications in neonatal infants with respiratory distress syndrome.
based on abstract	5	Searching for an immunogenetic factor that will illuminate susceptibility to non-tuberculous mycobacterial disease.<br>Genetics of community-acquired pneumonia.<br>Gene polymorphism in intensive care patients. Is the course of disease predetermined?<br>Genetic markers of predisposition to infectious complications in neonatal infants with respiratory distress syndrome.<br>Interleukin-1 receptor antagonist intron 2 variable number of tandem repeats polymorphism and respiratory failure in children with community-acquired pneumonia.

reviews	3	Systemic cytokine response in patients with community-acquired pneumonia.<br>Importance of severity of illness assessment in management of lower respiratory infections.<br>Genetics of sepsis and pneumonia.
other interleukin polymorphism	8	Genetic polymorphisms within tumor necrosis factor gene promoter region: a role for susceptibility to ventilator-associated pneumonia.<br>Interferon-gamma 874A>T genetic polymorphism is associated with infectious complications following surgery in patients with thoracic esophageal cancer.<br>The -308G/A polymorphism of TNF-alpha influences immunological parameters in old subjects affected by infectious diseases.<br>Alleles carried at positions -819 and -592 of the IL10 promoter affect transcription following stimulation of peripheral blood cells with Streptococcus pneumoniae.<br>Stroke, IL-1ra, IL1RN, infection and outcome.<br>Genetic polymorphisms within tumor necrosis factor gene promoter region: a role for susceptibility to ventilator-associated pneumonia.<br>Interleukin-1 receptor antagonist gene polymorphism in patients with multidrug-resistant Acinetobacter baumannii-associated pneumonia.<br>Haplotype of IL-8 -251T and 781C is associated with the susceptibility to respiratory syncytial virus.
articles with only patients	3	Variants at the promoter of the interleukin-6 gene are associated with severity and outcome of pneumococcal community-acquired pneumonia.<br>Association of IL-10 polymorphism with severity of illness in community acquired pneumonia.<br>Interleukin-10 haplotype associated with increased mortality in critically ill patients with sepsis from pneumonia but not in patients with extrapulmonary sepsis.
articles for duplicate search	1	Host genetic risk factors for community-acquired pneumonia.
